# 

**Published:** 1877-09

**Authors:** 


### Table of Contents.

### ORIGINAL COMMUNICATIONS.

Puerperal Insanity. *By V. H. Taliaferro, M.D., Atlanta, Ga* ...... 321
Broncho-Pneumonitis. *By R. B. Anderson, M.D., Roswell, Ga* ..... 329
Colo-Rectitis. *By J. G. Westmoreland, M.D., Atlanta, Ga* ......... 332
The Use of Bromide of Potassium Hypodermically. *By Wm. A. Adams, M.D., Bryan, Texas* ......... 335
Pleuro-Pneumonia, with Subsequent Scorbutus. *By D. W. Scott, M.D., Castlemount, Ga* ......... 336

### REPORTS OF SOCIETIES.

Transactions of Medical and Surgical Society of Baltimore .......... 337
Proceedings of the Columbus, O., Academy of Medicine ............ 339

### SELECTIONS.

Relative Value of Colchicum Root ............ 349
Suppurative Arthritis Following Acute Rheumatism ............ 252
Atmospheric Influences Controlling Inebriety ............ 354
Hæmoptysis ............ 358
Clinical Lecture on a Case of Pott's Disease of the Upper Cervical Vertebræ ............ 364
The Importance of Cincho-Quinine as a Remedy ............ 369

### EDITORIAL.

Opium Habit ............ 370
Our Department of Society Reports ............ 372
In Memoriam ............ 373
Editorial Correspondence ............ 373, 374
European Correspondence ............ 375, 279

### BIBLIOGRAPHICAL.

Micro-Photographs in Histology ............ 382
Pamphlets ............ 382

### EXCERPTA.

Death after Chloroform ............ 384
The Plaster-Paris Jacket ............ 384
How to Demonstrate the Presence of Carbonic Oxide in the Air of a Room ............ 384

